# Histopathologic Features of Antibody Mediated Rejection: The Banff Classification and Beyond

**DOI:** 10.3389/fimmu.2021.718122

**Published:** 2021-09-27

**Authors:** Lynn D. Cornell

**Affiliations:** Division of Anatomic Pathology, Department of Laboratory Medicine and Pathology, Mayo Clinic, Rochester, MN, United States

**Keywords:** antibody mediated allograft rejection, pathology and clinical outcomes, diagnostic criteria, kidney, transplant, sensitized, alloantibody

## Abstract

Antibody mediated rejection (ABMR) in the kidney can show a wide range of clinical presentations and histopathologic patterns. The Banff 2019 classification currently recognizes four diagnostic categories: 1. Active ABMR, 2. Chronic active ABMR, 3. Chronic (inactive) ABMR, and 4. C4d staining without evidence of rejection. This categorization is limited in that it does not adequately represent the spectrum of antibody associated injury in allograft, it is based on biopsy findings without incorporating clinical features (e.g., time post-transplant, *de novo* versus preformed DSA, protocol versus indication biopsy, complement inhibitor drugs), the scoring is not adequately reproducible, and the terminology is confusing. These limitations are particularly relevant in patients undergoing desensitization or positive crossmatch kidney transplantation. In this article, I discuss Banff criteria for these ABMR categories, with a focus on patients with pre-transplant DSA, and offer a framework for considering the continuum of allograft injury associated with donor specific antibody in these patients.

## Introduction

Since its initial meeting in 1991, the Banff classification for allograft pathology has become the most commonly used classification system for renal allograft pathology. Pathologists, HLA laboratory directors, nephrologists, surgeons, and researchers gather every other year to discuss recent advances in the field and to revise the classification system, and a meeting report with consensus opinion is published. The Banff schema first recognized antibody mediated rejection (ABMR) as a diagnostic entity in 2001 (published in 2003), making use of the complement split product C4d as an immunophenotypic marker of ABMR on biopsy (1). The ABMR criteria have been revised over the years, and continue to be revised, as knowledge progresses.

## Overview of Clinical Categories of ABMR and Banff ABMR Categories

ABMR can show a wide variety of clinicopathologic features, from hyperacute rejection with primary graft nonfunction, to early acute ABMR in positive crossmatch (+XM) kidney transplants, to progressive graft dysfunction or proteinuria years post-transplant. ABMR features are also seen commonly on protocol biopsies from patients who have preformed (pre-transplant) DSA; these patients have stable graft function and no proteinuria (2). There are additional clinicopathologic features in kidney transplant patients with *de novo* DSA, immunosuppressive medication nonadherence, or combined cellular rejection and ABMR (3–5). The challenge with any classification system is to fit a wide variety of potential clinicopathologic features into a reasonable number of distinct diagnostic categories, which ideally would have specific corresponding treatment protocols.

As of 2019, the Banff schema recognizes four diagnostic categories: 1. Active ABMR, 2. Chronic active ABMR, 3. Chronic (inactive) ABMR, and 4. C4d staining without evidence of rejection, The first category, “active ABMR”, requires 3 diagnostic criteria: histologic evidence of acute tissue injury, evidence of current or recent antibody interaction with the endothelium (usually C4d), and serologic evidence of DSA (although C4d staining or validated transcripts may substitute for DSA). Histologically, microvascular inflammation (MVI), also known as capillaritis, qualifying for this category is a glomerulitis (g) score + peritubular capillaritis (ptc) score of 2 or greater. Other acute tissue injury patterns are acute tubular injury, thrombotic microangiopathy, and less commonly arterial lesions of endothelialitis, fibrinoid necrosis, or transmural inflammation. Chronic active ABMR has a similar three criteria, but with histologic evidence of chronic tissue injury, such as transplant glomerulopathy (TG) attributable to ABMR. Chronic (inactive) ABMR shows histologic evidence of chronic tissue injury, but without capillaritis and without C4d deposition in peritubular capillaries.

The final category is peritubular capillary C4d staining without evidence of rejection, previously referred to as “accommodation” (6). This category primarily applies to ABO blood group incompatible transplants, which show positive C4d staining in even 80% of protocol biopsies and the staining does not correlate with peritubular capillaritis (7, 8). Despite positive C4d staining, ABO blood group incompatible kidney transplants show the same rate of capillaritis as conventional kidney transplants (8). Although some protocol biopsies in patients with +XM (anti-HLA DSA) transplants show positive C4d staining and no histologic evidence of tissue injury, and thus qualify for the category of accommodation, the state of accommodation in patients with anti-HLA DSA is likely temporary and unstable (8).

## Early Acute ABMR in +XM Kidney Transplants

The Banff 2019 category of “active” ABMR (previously known as “acute” or “acute/active” ABMR) by itself encompasses a wide variety of clinicopathologic features. Patients undergoing +XM kidney transplants are at increased risk of early acute ABMR within the first month post-transplant (9, 10). This type of ABMR is associated with very high serum DSA levels (measured at the time of biopsy), C4d deposition in peritubular capillaries, and graft acute tissue injury with thrombi and acute tubular injury (11). Clinically, although uncommon, this type of acute ABMR has been difficult to treat (11, 12). There is a high rate of graft loss if not recognized and treated (13, 14). More recently, early acute ABMR has been treated with the terminal complement inhibitor eculizumab (15). The current standard of care for treatment of this particular type of acute ABMR involves plasmapheresis, intravenous immunoglobulin, and corticosteroids, and adjunctive therapies of complement inhibitors, rituximab and splenectomy may be considered (16).

**Figure 1:**
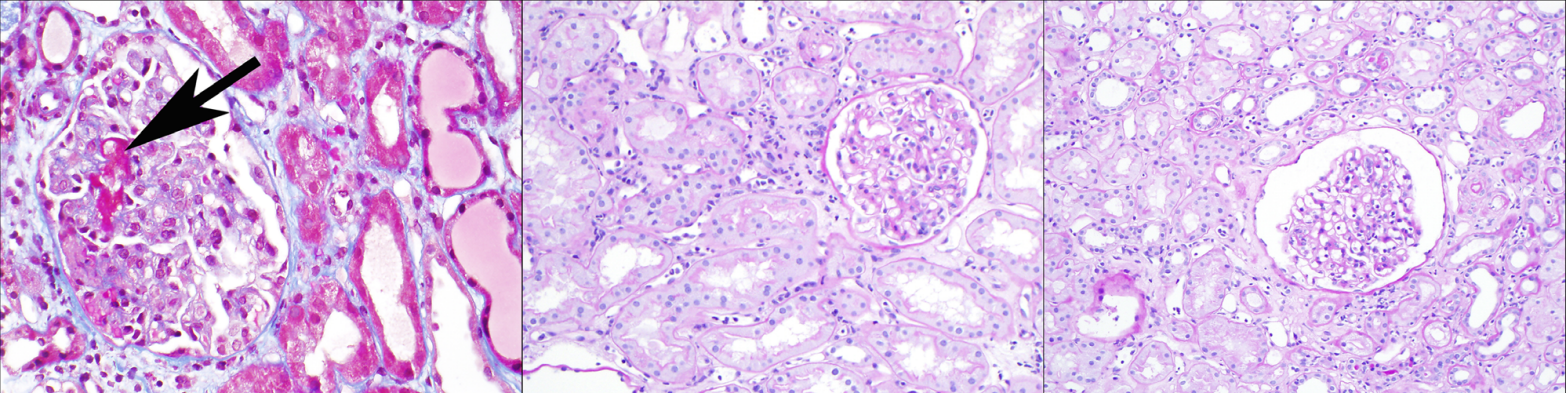
Three clinicopathologic phenotypes of ABMR, one Banff diagnosis: In the left panel (Masson trichrome stain), a patient 2 weeks after positive-crossmatch kidney transplant has acute kidney injury; the biopsy shows glomerular thrombi, acute tubular injury, and minimal capillaritis, and diffuse C4d deposition in peritubular capillaries. In the middle panel (periodic acid Schiff stain), a patient 4 months after positive-crossmatch kidney transplant has stable graft function and undergoes protocol biopsy; the biopsy shows glomerulitis and peritubular capillaritis; C4d staining is negative. In the right panel (periodic acid Schiff stain), a patient 2 years after kidney transplant has stable graft function and undergoes protocol biopsy; the biopsy shows glomerulitis and peritubular capillaritis; C4d staining is negative. All of these biopsies pictured would be assigned the Banff diagnosis of “active ABMR”, although clearly the clinical settings are different.

Histologically, these cases of early acute ABMR with high serum DSA levels often show an “ATN-like” (acute tubular necrosis) phenotype (Banff “grade 1”) with few marginated inflammatory cells (particularly neutrophils) in glomerular and peritubular capillaries, along with glomerular thrombi (see **Figure 1**, left panel). Some cases do show moderate microvascular inflammation (g + ptc >/=2), particularly when the pathologist is aware of the clinical consideration of acute ABMR as a diagnostic possibility with therapeutic implications (Banff “grade 2”) (11, 17, 18). These early acute ABMR biopsies are essentially always C4d positive, and C4d positivity correlates with the serum DSA level (11). It should be noted that the capillary inflammation does not mirror the clinical severity of this ABMR manifestation: a higher Banff capillaritis score does not indicate a more severe acute rejection phenotype. In this way, ABMR is different from acute cellular rejection, tubulointerstitial type (Banff grade IA or IB or borderline rejection), where more extensive inflammation with a higher Banff i or t score reflects a more severe rejection (19).

As these biopsies show evidence of complement activation, colleagues at Mayo Clinic conducted a pilot study of +XM kidney transplant recipients using a terminal complement inhibitor, eculizumab, to prevent early acute ABMR (20). Compared to historical +XM control patients, there was a marked reduction in the rate of early acute ABMR: overall, 41% (21/51) of controls developed early acute ABMR, compared to 7.7% of patients (2/26) in the eculizumab group (p=0.0031). Moreover, of those who developed high DSA levels within the first month post-transplant, and thus were at risk for early acute ABMR, 100% (20/20) of the control patients developed early acute ABMR, compared to 2/13 (15%) of the eculizumab group (p<0.0001). Both eculizumab treated and control patients in this group with high DSA (at the time of biopsy) showed C4d deposition in peritubular capillaries. Eculizumab is a terminal complement inhibitor, inhibiting the complement cascade downstream of C4d, and so even patients with effective terminal complement inhibition would be expected to show C4d deposition on the kidney biopsy. [These two patients who did develop early acute ABMR while receiving eculizumab responded well to treatment with plasmapheresis alone; further investigation of these patients’ serum suggested an IgM anti-HLA DSA, which likely responds differently to plasmapheresis treatment (21)] The striking results of this study suggests that this early acute ABMR in +XM patients is complement-mediated, rather than simply “antibody-mediated” as the name implies. I believe we should recognize this distinct clinicopathologic entity with its own name, such as early acute antibody and complement-mediated rejection in patients with preformed DSA.

## Active (Smoldering) ABMR

After the first few months post-transplant, patients with pre-transplant DSA may develop a more indolent form of ABMR, characterized histologically by mild to moderate capillaritis involving the glomeruli, peritubular capillaries, or both (“microvascular inflammation”, or MVI). These findings are commonly observed on protocol biopsies from patients with normal graft function and no proteinuria, ranging from 3 months to 5 years post-transplant (8, 22–24). This pattern of injury, in the absence of graft dysfunction, is sometimes referred to as “subclinical ABMR”. C4d may be focally positive (more common) or diffusely positive, and ranges from approximately 50% to <20% (less commonly positive on 5 year post transplant protocol biopsies) (8, 24). These changes are associated with later development of TG, and the risk of TG is greater in patients with C4d positivity on biopsy. MVI also commonly occurs in conjunction with chronic ABMR lesions, such as TG, once they have developed.

It is unclear how patients with MVI on biopsy, with or without early TG, should be treated. Since C4d is generally not diffusely positive, we can hypothesize that: (1) the corresponding serum DSA levels are low, and thus likely not responsive to plasmapheresis; and (2) the mechanism of injury may not be complement-mediated, at least not in the way early acute ABMR is complement-mediated. Supporting (2), a subset of patients showed endothelial injury changes and developed MVI, and even TG, even while receiving the terminal complement inhibitor eculizumab (22, 25). Treatment of this lesion is under investigation in clinical trials. Also, prevention, including through kidney donor paired exchange programs and “low positive” crossmatch transplantation, will help to reduce graft loss due to chronic ABMR (12).

**Figure 2:**
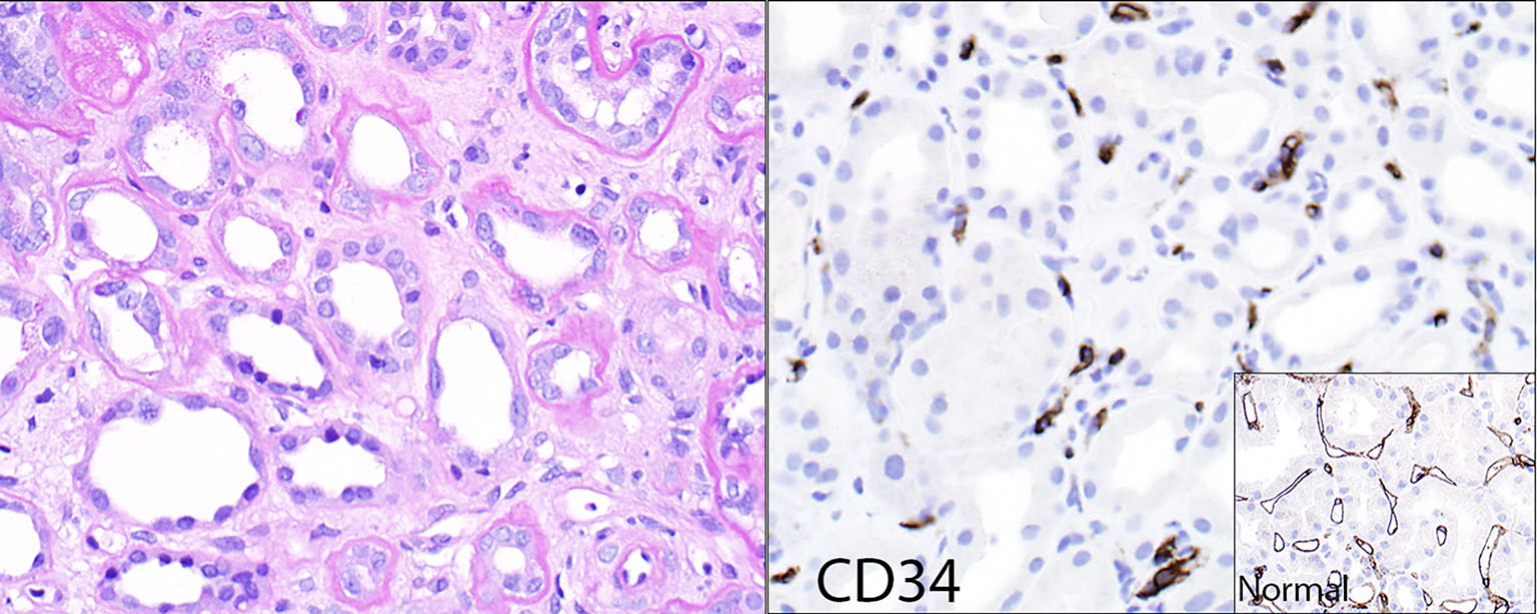
Loss of peritubular capillaries. Some transplants with chronic ABMR show such significant loss of peritubular capillaries that the capillaries simply no longer exist to show capillaritis. The left panel (hematoxylin & eosin stain) shows minimal peritubular capillaritis; the right panel shows the endothelial marker CD34 of the same case, revealing remnants of peritubular capillaries that were presumably destroyed by rejection. The insert shows a CD34 stain of a reference kidney transplant case without peritubular capillary loss.

According to the Banff classification, MVI (with the threshold of g + ptc >/= 2) is an “active” ABMR lesion. The current Banff schema thus may place some cases of early acute ABMR in the same category as a 2 year post-transplant protocol biopsy with mild glomerulitis and peritubular capillaritis and is C4d negative. (See **Figure 2**) Early acute ABMR is an aggressive form of acute ABMR with specific treatment protocols; while the latter, which we could term “smoldering ABMR”, appears to be more indolent and would not respond to the same treatment (14). It is important to recognize that the degree of capillaritis does not necessarily correspond to the severity or “activity” of the rejection. Incorporating time post-transplant, preformed versus *de novo* DSA status, and other clinical information such as acute kidney injury or protocol biopsy would inform pathologists and nephrologists as to a better classifying diagnosis along this spectrum of antibody associated disease.

## Chronic ABMR

Chronic active ABMR occurs when one or more of the “chronic” features are present on a biopsy: TG, peritubular capillary basement membrane multilayering (PTCBMML, also known as peritubular capillaropathy), or transplant arteriopathy. Of these, TG is the best characterized lesion of chronic ABMR. By definition, TG is a pattern of injury with glomerular basement membrane duplication in the absence of subendothelial immune complex deposits. TG is most commonly due to chronic ABMR, but the pattern may also be related to chronic thrombotic microangiopathy and/or hepatitis C infection (26). Biopsies with TG due to ABMR may show negative, minimal, focal or multifocal, or diffuse C4d deposition in peritubular capillaries. Approximately 20-40% of biopsies with TG due to ABMR show positive C4d staining, but this percentage will vary widely and depend on many factors – protocol versus indication biopsy, +XM kidney transplant status, preformed versus *de novo* DSA timing of the biopsy post-transplant, presence of concurrent cellular rejection, and immunosuppressive medication non-adherence (27–29). While TG overall has a bad prognosis, the prognosis is variable. TG does have a worse prognosis when peritubular capillaries are C4d positive – likely reflecting a more “active” rejection phenotype with higher DSA levels and complement activation (27, 28). Other clinical, laboratory, and biopsy features besides C4d may be helpful in determining the “activity” and prognosis of TG for a particular patient, and potential treatment or enrollment in clinical trials. These may include time post-transplant, DSA antibody level and type (e.g., anti-HLA class I or II), proteinuria, degree of capillaritis, peritubular capillaropathy (PTCBMML), concurrent cellular rejection, and medication non-adherence. Future directions in ABMR categorization would include validation of activity and chronicity scores such as for these features.

The current Banff classification lumps TG cases together in one diagnostic category, but TG has a variable prognosis. An outcomes-based approach may be used: a study looked at an “archetype” classification, incorporating clinical, histologic, and immunologic features, which placed patients with TG into different prognostic or outcomes groups (30). These clinicopathologic archetypes may be useful to incorporate into the current biopsy-centric diagnosis, for TG now and at some point for other manifestations of ABMR.

The Banff schema also has a category of chronic (inactive) ABMR. This category may be theoretical, since DSA and its resultant effects do not seem to disappear at any point post-transplant, at least with current therapies (or rather, lack of effective therapies). Biopsies from patients with advanced chronic kidney disease due to ABMR generally show capillaritis as long as capillaries exist. Occasional transplants show such significant loss of peritubular capillaries that the capillaries simply no longer exist to show capillaritis (**Figure 2**).

## Application of the Banff ABMR Criteria in Clinical Practice

At the 2013 Banff conference in Comandatuba, Bahia, Brazil, a new Banff Working Group was formed, the “Clinical and Laboratory Assessment of Highly Sensitized Patients Working Group”, later referred to as the “Antibody-Mediated Injury Working Group” (31). This Working Group, in which the author (LDC) participates, conducted a survey of clinical practices related to antibody-mediated injury (32). This survey included six clinicopathologic ABMR scenarios and corresponding kidney biopsies, meant to reflect a broad spectrum of injury, including acute/active ABMR and chronic ABMR, and mixed cellular and antibody-mediated rejection. Both pathologists and clinicians (nephrologists/transplant surgeons) responded. There was a discrepancy between the reference (Banff assigned) diagnosis and the pathologist or clinician diagnosis overall approximately 30% of the time. Moreover, this discrepancy influenced treatment decisions. We concluded that the term “acute” in “acute/active ABMR” is confusing, and consequently it was removed from the Banff 2017 schema. We also found that clinicians often failed to recognize the “chronic” elements of ABMR, such as transplant glomerulopathy (TG). They were more likely to consider a diagnosis of chronic active ABMR if the C4d stain was negative, even if there was no TG, PTCBMML, or interstitial fibrosis/tubular atrophy.

Our Working Group then conducted a follow up survey in 2019 of pathologists and transplant clinicians to address additional questions raised by the first clinical practices survey (33). We found that most (97%) respondents said they used the Banff ABMR classification at least sometimes; however, only 19% of pathologists and 41% of nephrologists/surgeons always had DSA results when the kidney biopsy was interpreted. In fact, 18% of 99 pathologists surveyed responded that they *never* had DSA testing results when interpreting the kidney transplant biopsy. Recall that DSA information is central to Banff ABMR diagnosis, although not necessarily required for all kidney transplant biopsy diagnoses. 40% of pathologists agreed that the time post-transplant influenced their ABMR diagnosis and 58% agreed that they consider allograft dysfunction in considering a diagnosis. One of the main concerns identified was the dichotomous nature (active and chronic active ABMR) of the Banff ABMR categories. These results reflect the reality of renal pathology practice, and the recognition that the Banff schema does not reflect the full range of ABMR.

Another concern that some survey respondents raised was that the Banff ABMR classification changed too frequently for practical use (33). A recent paper from J Callemeyn and colleagues evaluated kidney transplant biopsies and classified their histologic features, C4d staining status, and DSA status into different ABMR categories by the Banff 2001, Banff 2013, and Banff 2017 classifications (34). The authors found – from the same set of biopsies – that there was significant reclassification of the biopsies, between no ABMR, “suspicious for ABMR”, and ABMR. The Banff 2013 classification resulted in a marked increase in the number of biopsies classified as ABMR or suspicious for ABMR, and the 2017 classification adjusted this downward. Of course, it was by design that reclassification occurred, as the Banff schema was changed in an attempt to reflect updated knowledge of the ABMR process. But with potential future iterations of the Banff ABMR criteria, this kind of analysis could be used to gauge clinical impact, including incorporating clinical features such as graft dysfunction and time post-transplant, and molecular markers.

## Reproducibility of Histologic Scores, ABMR Grading, and Incorporation of Clinical Information

Besides determining the “ideal” histologic criteria and cut-off points for different categories of ABMR, the Banff schema should also take into account problems with reproducibility of the Banff scores. Recognizing problems with the Banff “antibody” scores reflecting microvascular inflammation and transplant glomerulopathy as currently defined, a number of studies have looked at reproducibility and methods to improve the scoring and the relation between antibody scores and graft outcome (35–38).

In a study we conducted at Mayo Clinic, colleagues and I reviewed a set of biopsies from patients with preformed DSA, and evaluated them for the “antibody” scores of g, ptc, and cg (transplant glomerulopathy) (39). This study included 6 renal pathologists from 3 Mayo Clinic sites. We found poor agreement for a specific score by any 2 pathologists, ranging from 45-66% agreement for g, 45-67% for ptc, and 54-81% for cg. Furthermore, in 22% of cases, review of the same slide by a different pathologist would result in a different Banff ABMR diagnosis based on histology alone (no ABMR, active ABMR, or chronic ABMR). An improvement analyzed in this paper could be made by a “majority rules” approach with review by three pathologists.

A randomized, multi-center clinical trial was conducted to determine the safety and efficacy of the C5 complement inhibitor drug, eculizumab, to prevent early acute ABMR in positive-crossmatch kidney transplant patients, following an initial pilot study at Mayo Clinic with positive results (18, 20). The primary endpoint of the multi-center study was a composite of biopsy-proven acute ABMR and graft loss or death. The initial results, surprisingly, showed no significant difference in the rate of acute ABMR among patients who received eculizumab versus the standard of care. How could this be? First, “positive crossmatch” was defined differently at different participating institutions, and so patients likely had different degrees of risk for early acute ABMR (early acute humoral rejection). Second, in the initial analysis, only Banff 2007 ABMR types/”grades” II and III were included (presumably as an indicator of rejection severity), as assessed histologically by a central pathologist. As discussed above, early acute ABMR often shows an “ATN-like” histologic pattern, which is ABMR type or “grade” I and would not qualify for the rejection endpoint in this study. The data were re-analyzed to include ABMR grades I, II, and III, and then there was a significant difference between the eculizumab and standard of care groups, the latter showing a higher rate of rejection. Third, the biopsies were reviewed initially by the local pathologist, who had knowledge of the clinical setting (e.g., graft dysfunction, DSA levels, communication with a clinician about the possibility of acute ABMR); the local pathologist rendered a diagnosis of acute ABMR (particularly grades I and II) more often than the central pathologist. The central pathologist was removed from the clinical setting, and rendered a diagnosis based on histology only, at least in the initial analysis. Conclusions we can draw from this study with regards to the Banff ABMR classification are: 1) Banff grading and categorization of the biopsies affected the design and outcome of this trial; 2) Histologic “grade” does not necessarily imply severity or treatment responsiveness in acute ABMR; and 3) Access to clinical information and laboratory results leads to a higher rate of rejection diagnosis in sensitized kidney transplant recipients. As more drugs are being developed and tested for kidney transplant patients with ABMR, there is an urgent need to re-evaluate biopsy-based clinical trial entry and endpoint criteria independent of the current Banff classification.

Like all of medical renal pathology, the renal transplant biopsy diagnosis is a clinicopathologic diagnosis. The renal pathologist identifies the histologic pattern, and then the disease, and then ideally the cause of that disease. For example, a biopsy shows a pattern of acute tubular injury with calcium oxalate crystals, the disease is acute oxalate nephropathy, and the cause is enteric hyperoxaluria due to Roux-en-Y gastric bypass. Just as renal pathologists use a clinical history of diabetes to diagnose diabetic glomerulosclerosis, a history of a vasculitic rash to diagnose Henoch-Schoenlein purpura, a history of antibiotic exposure to diagnose acute allergic interstitial nephritis, or a history of autoimmune pancreatitis and sialadenitis to diagnose IgG4-related tubulointerstitial nephritis, we similarly use the clinical history and laboratory results to make a diagnosis on transplant biopsies. While there is value in reproducible histologic scoring systems with outcomes measures (such as the Oxford classification for IgA nephropathy), we should not expect any classification system to assign an accurate diagnosis based on histology alone, and particularly in the transplant, where most “native” kidney diseases may occur, and in addition, those diseases specific to the transplant may also occur.

## ABMR Continuum

**Figure 3:**
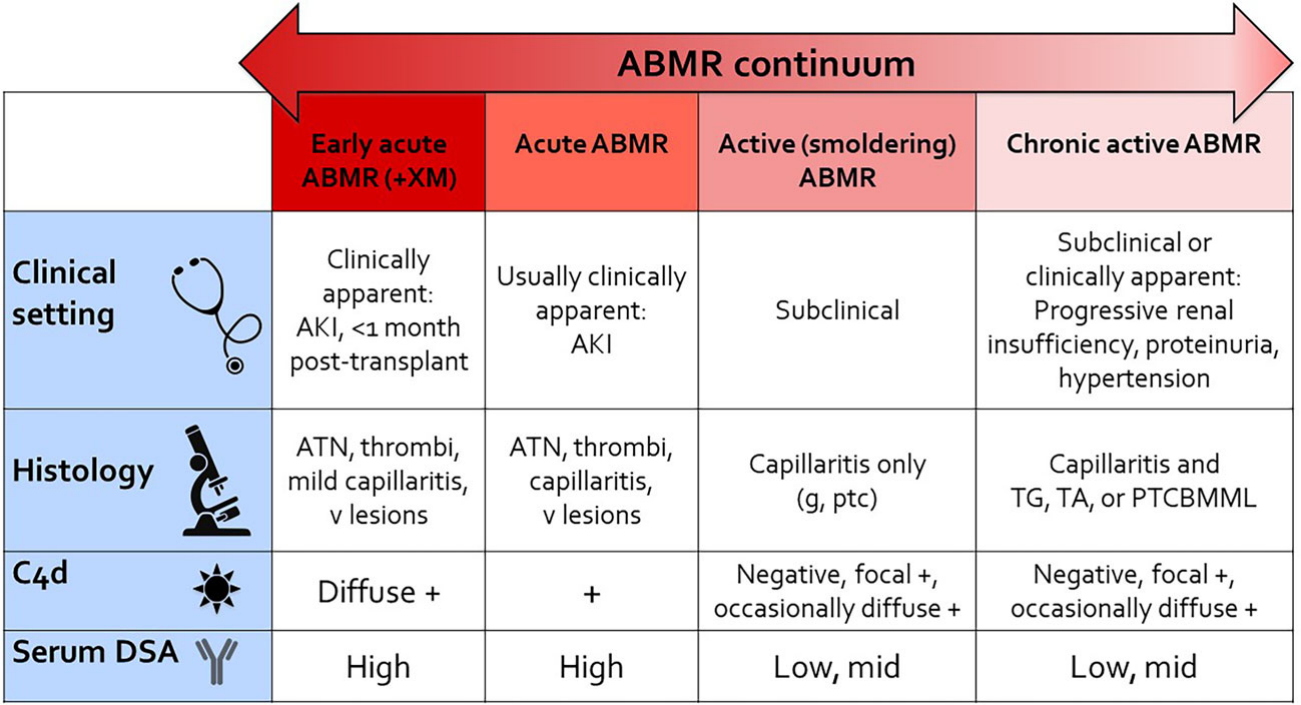
ABMR continuum. This schematic provides a reference for thinking about the continuum of “pure ABMR” in kidney transplant recipients with preformed DSA, as detailed in this article. Not included in the figure is combined ABMR and T cell mediated rejection in patients with *de novo* DSA and under-immunosuppression (iatrogenic or due to nonadherence). AKI, acute kidney injury; ATN, acute necrosis/injury; g, glomerulitis; ptc, peritubular capillaritis; v lesions, Banff vascular lesions (endothelialitis, fibrinoid necrosis of vessels); TG, transplant glomerulopathy; TA, transplant arteriopathy; PTCBMML, peritubular capillary basement membrane multilayering (by electron microscopy); +XM, positive crossmatch.

**Figure 3** shows a framework for considering the spectrum of ABMR, including some existing Banff categories, ranging from the most “acute” or active rejection on the left to the more chronic and progressive changes on the right. Clinically, positive crossmatch patients with early acute humoral rejection within the first month post-transplant present with acute kidney injury, show more “acute” features histologically, show diffuse C4d deposition in peritubular capillaries associated with high serum DSA levels. Acute ABMR shows similar features but may not have as severe a phenotype, and may occur later post-transplant. Active (smoldering) ABMR is subclinical, shows capillaritis only, and is seen on protocol biopsies from patients with DSA. Chronic active ABMR is subclinical and seen on protocol biopsy, or may be clinically apparent, and shows “chronic” features on biopsy. C4d staining and serum DSA levels may be variable depending on disease activity at the time of biopsy. This schematic shows only “pure” ABMR, as is seen in positive crossmatch or sensitized patients, and does not take into account combined ABMR and cellular rejection, which adds an additional level of complexity. While it is most convenient if patients have a known history of DSA at some point, detectable circulating DSA is not always present, or this information is not available at the time of biopsy. I believe the previous Banff designation of “suspicious” for ABMR would be useful in these situations when the allograft biopsy shows histologic features otherwise typical of ABMR.

## Future Directions in ABMR Diagnosis

What are potential ways to address many of these concerns? First, the Banff diagnostic categories should recognize and incorporate the broad range of clinicopathologic phenotypes of ABMR. Different patterns of rejection occur in different settings (e.g., transplantation in sensitized patients) and at different times post-transplant, and so the clinical setting is useful for a more accurate histopathologic diagnosis. In addition to the ABMR spectrum in **Figure 3**, a range of markers of “activity”, including C4d positivity and ultrastructural endothelial activation, for example, and “chronicity”, including TG, could be incorporated into activity and chronicity scores, akin to the modified NIH lupus activity and chronicity indices (40). Such an ABMR index would need to be tested and validated, such as with the Callemeyn biopsy set, ideally including outcomes measures. Testing and validation of histologic and immunophenotypic markers, including capillaritis, individually and in combination could eventually clarify the meaning of confusing terms such as “acute”, “active”, and “chronic”.

An “archetypes” approach, as has been evaluated in TG (30), could be taken with other antibody-associated patterns of injury. At some point, molecular markers may be incorporated into diagnosis (41, 42), although these would need to be validated and also widely available for clinical use. Clinical trials may be a starting point for incorporation of new molecular markers as classifiers of ABMR categories. Another approach may be to incorporate digital pathology and machine learning into the diagnostic schema, perhaps including immunohistochemical stains to quantify the capillary inflammation and endothelial reactive changes. Again, such tests would need to be validated. Availability of resources is a challenge for widespread incorporation of these techniques, although clinical trials could serve as a starting point.

## Conclusion

ABMR in the kidney shows a wide range of clinicopathologic features that are not adequately represented in the current Banff diagnostic classification. For current clinical practice and clinical trials, we need to re-assess the usefulness of the current ABMR categories, and more accurately reflect the spectrum of antibody-associated injury and their prognostic categories.

## Data Availability Statement

The original contributions presented in the study are included in the article/supplementary material. Further inquiries can be directed to the corresponding author.

## Author Contributions

The author confirms being the sole contributor of this work and has approved it for publication.

## Conflict of Interest

The author declares that the research was conducted in the absence of any commercial or financial relationships that could be construed as a potential conflict of interest.

## Publisher’s Note

All claims expressed in this article are solely those of the authors and do not necessarily represent those of their affiliated organizations, or those of the publisher, the editors and the reviewers. Any product that may be evaluated in this article, or claim that may be made by its manufacturer, is not guaranteed or endorsed by the publisher.
